# Vancomycin-resistant *vanB*-type *Enterococcus faecium* isolates expressing varying levels of vancomycin resistance and being highly prevalent among neonatal patients in a single ICU

**DOI:** 10.1186/2047-2994-1-21

**Published:** 2012-05-30

**Authors:** Guido Werner, Ingo Klare, Carola Fleige, Uta Geringer, Wolfgang Witte, Heinz-Michael Just, Renate Ziegler

**Affiliations:** 1 Unit FG13 Nosocomial Infections, Robert Koch-Institute Wernigerode, Wernigerode, Germany; 2 Institute for Clinical Hygiene and Infectiology, Hospital Nord der Stadt Nürnberg, Nuremberg, Germany

## Abstract

**Background:**

Vancomycin-resistant isolates of *E. faecalis* and *E. faecium* are of special concern and patients at risk of acquiring a VRE colonization/infection include also intensively-cared neonates. We describe here an ongoing high prevalence of VanB type *E. faecium* in a neonatal ICU hardly to identify by routine diagnostics.

**Methods:**

During a 10 months’ key period 71 *E. faecium* isolates including 67 *vanB*-type isolates from 61 patients were collected non-selectively. Vancomycin resistance was determined by different MIC methods (broth microdilution, Vitek® 2) including two Etest® protocols (McFarland 0.5/2.0. on Mueller-Hinton/Brain Heart Infusion agars). Performance of three chromogenic VRE agars to identify the *vanB* type outbreak VRE was evaluated (Brilliance^TM^ VRE agar, chromID^TM^ VRE agar, CHROMagar^TM^ VRE). Isolates were genotyped by *Sma*I- and *Ceu*I-macrorestriction analysis in PFGE, plasmid profiling, *vanB* Southern hybridisations as well as MLST typing.

**Results:**

Majority of *vanB* isolates (n = 56, 79%) belonged to a single ST192 outbreak strain type showing an identical PFGE pattern and analyzed representative isolates revealed a chromosomal localization of a *vanB2*-Tn*5382* cluster type. Vancomycin MICs in cation-adjusted MH broth revealed a susceptible value of ≤4 mg/L for 31 (55%) of the 56 outbreak VRE isolates. Etest® vancomycin on MH and BHI agars revealed only two *vanB* VRE isolates with a susceptible result; in general Etest® MIC results were about 1 to 2 doubling dilutions higher than MICs assessed in broth and values after the 48 h readout were 0.5 to 1 doubling dilutions higher for *vanB* VRE. Of all *vanB* type VRE only three, three and two isolates did not grow on Brilliance^TM^ VRE agar, chromID^TM^ VRE agar and CHROMagar^TM^ VRE, respectively. Permanent cross contamination via the patients’ surrounding appeared as a possible risk factor for permanent VRE colonization/infection.

**Conclusions:**

Low level expression of *vanB* resistance may complicate a proper routine diagnostics of *vanB* VRE and mask an ongoing high VRE prevalence. A high inoculum and growth on rich solid media showed the highest sensitivity in identifying *vanB* type resistance.

## Background

A recent Health Care report of the European Center for Disease Prevention and Control lists Enterococcus spp. as the second most prevalent ICU-acquired bloodstream and urinary tract infection pathogen [1]. Special problems are linked to multi- and vancomycin-resistant variants hardly to treat by standard antibiotic regimes. The reservoir of acquired vancomycin resistance is in the species *E. faecium*. Altogether eight types of acquired vancomycin resistance genotypes *vanA-vanN* are known in enterococci with *vanA* the worldwide most prevalent genotype followed by *vanB*[2]. VanB type resistance is characterized by resistance to vancomycin and susceptibility to other glycopeptides like teicoplanin since only the former antibiotic is capable of inducing the *vanB* resistance type. Levels of expression of vancomycin resistance are generally higher in VanA than in VanB type strains meaning MICs against vancomycin are several dilution steps higher in VanA (commonly 16–512 mg/L) than in VanB strains (4–64 mg/L). Low level vancomycin resistance expression especially in VanB strains may complicate performance of diagnostic assays assessing the resistance phenotype and predicting the corresponding genotype. During recent years, clusters of infections and colonizations with *vanB* genotype *E. faecium* increased in a number of European countries. Sweden experienced several outbreaks with *vanB* type VRE [3] and country-wide surveillance of VRE notified an increasing number of *vanB*-type VRE in low prevalence countries like France [4]. The PCR-determined *vanB* type ligase gene appears in three allelic variants with *vanB2* as the most frequent. The *vanB2* gene is part of similar “Integrative and Conjugative Elements” (ICE) of the Tn*5382*- and Tn*1549*-types and thus transferable as well [5]. Common *vanB* type clusters were most often chromosomally located as, for instance, described for the *E. faecalis* type strain V583 [6]. The recent outbreaks of VanB strains in Sweden were linked to *vanB2*-Tn*5382* elements located on transferable pRUM-like plasmids [7,8].

In Germany, different surveillance schemes exist in parallel assessing also numbers of clinical cases and VRE/enterococcal infections in general. Despite having different denominators and foci, they mainly report similar frequencies of vancomycin resistance among *E. faecium* from colonizations and infections in hospital patients being around 10–15% in recent years [9,10]. The *vanA* genotype was the most wide-spread for many years in Germany and clusters of infections with *vanB* type VRE remained exceptional; however, this has changed since 2008 [10]. In a background of similar levels of vancomycin resistance, data of a number of surveillance schemes showed a permanent decrease in frequency of teicoplanin resistance since 2008, a marker of VanB type resistance. This has been confirmed by a genotypic assessment of resistance genotypes and is represented in the growing number of VanB strains sent to the German Focal laboratory for enterococci; from 2009 to 2011 more *vanB* type VRE than *vanA* type VRE were received representing outbreaks or an ongoing high overall prevalence of infections and colonisations with *vanB* type *E. faecium* in more than 20 university hospitals countrywide (Klare et al., unpublished data). Here we report a molecular-epidemiological investigation of a cluster of *vanB* type VRE cases in neonatal patients of a single ICU during an 10 months’ period. VRE were identified by a non-selective stool sample screening and subsequent VRE identification/confirmation. From this key period altogether 71 VRE were subjected to a deeper molecular analysis.

## Methods

### Hospital setting

The neonatal ICU belongs to a hospital of tertiary care (2.300 beds) located in South-Western Germany. It consists actually of two units with one containing 16 beds in four rooms and an additional one containing 20 beds in 5 rooms. Unit 1 offers the possibility of mechanical ventilation; patients are regularly transferred between the two units. The setting is conceptually a mixed ward allowing also older, pediatric patients to be admitted into two of the rooms. The neonatal ICU is a so-called “Perineonatology level 1 Centre” with an annual number of about 50 neonatal patients with a low birth weight of <1.500 g.

### Patient setting, bacterial isolates and primary diagnostics

During a period of September 2008 until June 2009 altogether 598 patients attending a neonatal ICU were screened non-selectively for enterococci. Columbia Agar with sheep blood (COL SB; Oxoid, Wesel, Germany) was used to isolate enterococci from different clinical samples. *E. faecium* was identified by using standard microbiological methods including hydrolyzing esculin and growth in 6.5% NaCl and by API 20 Strep (bioMérieux, Nürtingen, Germany). Randomly chosen enterococcal isolates were subsequently tested for resistance to vancomycin by routine diagnostics using agar diffusion or Etest® Vancomycin (bioMérieux). Susceptibility interpretations followed the guidelines proposed by CLSI (S ≤4; I =8/16; R ≥32 mg/L). Vancomycin resistance genotypes (*vanA*, *vanB* or *vanC*) were determined by a PCR and Southern hybridization based assay (GenoType® Enterococcus, Hain Lifescience, Nehren, Germany). Discrepancies between phenotypic (susceptible) and genotypic (*vanB*-positive) results lead to the general agreement to test all isolates for *vanB* with an inhibition zone of ≤18 mm around a vancomycin disk by a genotypic method. Seventy-one pre-selected *E. faecium* isolates were sent for *vanB* type confirmation and clonal analysis to the German focal laboratory for enterococci at the Robert Koch Institute.

### Antibiotic susceptibility testing

For all 71 *E. faecium* isolates antibiotic susceptibilities were determined for 14 antibiotics as minimal inhibitory concentrations (MIC) using a microdilution method in cation-adjusted Mueller-Hinton broth according to international standards. We used the EUCAST clinical breakpoints when available; for other antibiotics we applied breakpoints derived from CLSI, DIN and based on other criteria (e.g., for high level ciprofloxacin resistance >16 mg/L [11]). MICs were classified as resistant (in mg/L) as follows: penicillin/ampicillin >8, vancomycin >4; teicoplanin >2, erythromycin >4, linezolid >4, tetracycline >4, rifampicin >0.5, chloramphenicol >16, tigecycline >0.5, daptomycin >4, gentamicin (high-level) >128, streptomycin (high-level) >512, quinupristin/dalfopristin >4. Etest for vancomycin was performed according to the recommendation of the manufacturer (bioMérieux). In brief, two different protocols were followed. First, a standard screening method with Mueller-Hinton agar and an inoculum equivalent to McFarland 0.5 and second, Brain Heart Infusion agar and an inoculum equivalent to McFarland 2.0 was used. The latter one is called Etest® macromethod and is suggested for a confirmation of a supposed vancomycin resistance phenotype. Values are read after incubation at 35°C for 24 and 48 h as recommended (Etest® application sheet for Enterococcus/VRE and vancomycin EAS009). *E. faecalis* ATCC29212 and *E. faecium* ATCC19434 were used as control strains. Performance of three commercially available, chromogenic VRE screening agars was evaluated; Oxoid Brilliance^TM^ agar VRE (Thermo Scientific Fisher, Wesel, Germany); chromID^TM^ VRE (bioMérieux) and CHROMagar^TM^ VRE (Mast Diagnostika, Reinfeld, Germany). Strains were streaked out on selective plates and incubated as recommended by the manufacturers. Growth as single colonies and with the equivalent colours was rated as a positive result.

### DNA isolation

Genomic DNA was prepared using a DNA extraction kit (DNeasy Tissue Kit; Qiagen, Hilden, Germany) according to the manufacturer’s instructions. An initial cell wall lysis step was added dissolving the cell pellet in TES buffer [10 mM Tris, 0.5 mM ethylene diamine tetra-acetic acid (EDTA), 10% sucrose (pH 8.0)] plus 10 mg/mL lysozyme (Roche Applied Science, Mannheim, Germany) followed by incubation at 37°C for 30 min. Plasmids were extracted according to an alkaline lysis protocol and subsequent phenol/chloroform-based purification as described recently [12].

### PCR

PCR was performed with a PCR master mix (Thermo Fisher Scientific; St. Leon-Rot, Germany) according to the manufacturer’s instructions. Exactly 0.5 μL of isolated genomic DNA (ca. 10 ng) and primers (200 nM each) were added. Amplification of fragments representing the *esphyl*_*Efm*_ and *vanA/B* genes was performed in a multiplex PCR as described elsewhere [12]. Subtyping of *vanB* ligases and cluster types was done as described recently [13,14]. Primers vanB-L1: 5’-GTTTGATGCAGAGGCAGACGACT and vanB-L2 5’- ACAAGTTCCCCTGTATCCAAGTGG were used to amplify a 5,959 bp product using the Expand Long Template PCR system and conditions set by the manufacturer (Roche Applied Science, Mannheim, Germany). Long PCR products were subsequently digested with *BspH1* and *DraI* for 2 h at 37°C and resolved in 0.8% agarose gels. Plasmid replicase genes were amplified as described [15,16]. PCR for IS*16* was performed as described [17]. The following strains and plasmids were used as positive control samples: plasmid pRUM (IS*16, rep*_*17*_*family*), plasmid pLG1 (*hyl*_*Efm*_, repA-N family, new subtype), plasmid pIP816 (*vanA*; *E. faecium* BM4147), *E. faecium* U0317 (*esp*), and *E. faecalis* V583 (*vanB*) and E. faecalis RE25 pRE25 (inc18 rep_2_ family). *E. faecalis* OG1RF served as a negative control sample for all PCR assays.

### Mating experiments

Altogether nine ST192 strains (UW7606, UW7609, UW7611, UW7612, UW7813, UW7819, UW7835, UW7842, UW7845) were used as donors in in vitro filter-mating experiments. The rifampicin- and fusidic acid-resistant *E. faecium* strain 64/3 was used as a recipient. Transconjugants were selected on BHI agar supplemented with rifampicin (30 mg/L), fusidic acid (20 mg/L) and vancomycin with various concentrations according to the MIC of the donor strain (2, 4, 8 mg/L). The mating protocol was performed and mating rates were calculated as described recently [18]. Plates were incubated at 37°C up to 48 h. Supposed transconjugants were grown on selective plates and analyzed phenotypically (antibiotic susceptibilities) and genotypically (PCR-based marker genes and PFGE).

### PFGE analyses

Genomic DNA for PFGE analysis was isolated and treated as described recently [18]. The agarose gel concentration was 1%, the CHEF-DR III apparatus (Bio-Rad Laboratories, Hercules, CA, USA) was used for PFGE. *Sma*I-digested *Staphylococcus aureus* NCTC 8325 was used as a molecular mass standard on all PFGE gels. Genomic DNA of the *E. faecium* isolates was digested with *Sma*I. The ramped pulsed times were as follows: 1 – 11 s for 15 h and 11 – 30 s for 14 h at 14°C. Digestion of genomic DNA with I-*Ceu-I* linearises chromosomal DNA by recognizing the six rDNA operons in *E. faecium* revealing six chromosomal bands in PFGE. Genomic DNA was digested with I-*Ceu-I* for 16 h at 37°C. The ramped pulsed times for I-*Ceu-I* gels were 5 – 30 s for 22 h at 14°C [12].

### Southern hybridizations

Southern hybridization experiments were done as described elsewhere using a PCR-generated digoxigenin-labelled *vanB* probe (DIG High Prime; Roche Applied Science), hybridization chemicals and equipment from commercial kits and according to recommendations of the manufacturer (Roche Applied Science). Immunological detection was done as recommended using a chemiluminescent probe (CDP-Star^TM^, Roche Applied Science) and several readouts were taken at 10, 30, 60 and 120 min in a chemi-imager from Bio-Rad (Chemidoc XRS, Bio-Rad Labs., Hercules, US).

### MLST and DNA sequencing

PCRs amplifying the seven loci used for MLST were done according to the reference (http://efaecium.mlst.net/). Sequencing reactions were performed according to the manufacturer’s recommendations for cycle sequencing of PCR products (Life Technologies/Applied Biosystems, Germany). Sequence files were read, evaluated, aligned and compared to the reference set of alleles using sequencing software Lasergene 8.0 from DNA-STAR (SeqMan 8.0; EditSeq 8.0), TraceEditPro v. 1.1.1 from Ridom (http://www.ridom.de), and via the official MLST webpage (http://efaecium.mlst.net/).

### Statistics

Statistical analyses were performed with software package EpiCompare 1.0 (Ridom).

## Results

### Primary diagnostics

During a period of September 2008 until June 2009 almost 600 patients attending a neonatal ICU were screened non-selectively for enterococci and altogether 80 VRE carriers were primarily diagnosed. Seventy-one isolates including 67 *vanB*-type VRE and 4 vancomycin-susceptible *E. faecium* were available for a detailed molecular analysis*.* The 67 *vanB* isolates were from 61 patients, four *vanB* VRE were from patients’ surroundings (control monitor, incubator holding, incubator mattress) and from one patient three *vanB*-type *E. faecium* were sampled within 23 days*.* All but one patients were between 0–11 months old (a single case was 6 years old). The median age was less than one month; the majority of babies had been transferred into the neonatal ICU directly after birth where they were intensively cared.

### Infection control measures

Since bundle measures are known to be effective especially in containing outbreaks with VRE [19] various intensified infection prevention measures including an intensified disinfection procedure were established soon after the first cluster of VRE cases was identified. The catalogue of all these measures should only be mentioned here in brief: (a) intensified screening for VRE to define the extent of the scenario; (b) environmental sampling to identify contaminated surfaces as possible spreading sources; (c) introduction of barrier precautions for medical personal (gowns, gloves, masks, etc.); (d) permanent infection prevention and control training focussing on preventing environmental contamination and spread of pathogens; (e) interdisciplinary results’ discussion with medical staff and doctors; (f) repeated updating of an adequate antibiotic therapy; (g) rectal screenings of newborns directly after delivery to identify a possible introduction of VRE into the neonatal ICU from other wards/outside (see below); (h) extended barrier precautions also for family members of the neonatal patients. Comprehensive epidemiological and infection control analyzes in the described neonatal setting were neither able to identify the source of the *vanB* type strains nor reliable routes of transmission.

Recurrent introduction of VRE by patient admissions into the neonatal ICU was evaluated as a possible risk factor for a high VRE prevalence. Most neonatal patients were directly admitted after delivery to the neonatal ICU (median age of 0 months). An one-month screening (rectal swabs) of 100 neonates directly after delivery and before attending the neonatal ICU did not reveal any positive VRE result (but 56 with bacterial growth in general) excluding mothers and other family members as potential sources (not described in details).

After introducing extended barrier precautions also for family members of the neonates in August 2009, VRE cases dropped continuously (09-12/2008: n = 58; 2009: n = 71; 01-09/2010: n = 13) and from 2011 on, new VRE cases were not noticed.

### Clinical case descriptions

A retrospective analysis of clinical data of all identified neonatal VRE cases between 2008 and 2010 (n = 158) revealed valid and retrospectively analyzable data for about two third of them (n = 103). Vast majority of VRE cases were colonizations (anal/rectal, skin, throat); three babies were diagnosed with an enterococcal infection: one with a catheter sepsis, one with a necrotising enterococolitis and one with high enterococcal yields clinically linked to a ventilator-associated pneumonia (role of *Enterococcus* questionable); nevertheless, ten neonatal patients received linezolid therapy due to a suspected VRE infection.

### Antimicrobial susceptibility patterns

For all 71 *vanB* VRE isolates available for a further analysis the MICs were determined. All *E. faecium* isolates were resistant to ampicillin and susceptible to teicoplanin, chloramphenicol, linezolid and daptomycin. Resistances to other antimicrobials appeared as follows: erythromycin in 67 (94%), ciprofloxacin (high-level) in 64 (90%), gentamicin (high-level) in 59 (83%), streptomycin (high-level) in 23 (32%; but 40 [56%] had an MIC of 512 mg/L), vancomycin in 33 (46%), tetracycline in 7 (10%) and quinupristin/dalfopristin 3 (4%) and tigecycline 1 (1%; MIC of 1 mg/L).

## PCR results

**Figure 1:**
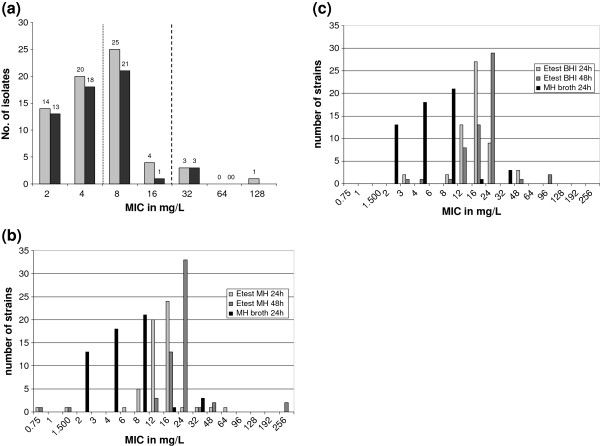
**Distribution of vancomycin MICs.** (**a**) Vancomycin MICs of the 67 *vanB* type VRE (light grey) and of the subset of 56 *vanB* outbreak strains (dark grey) determined by microdilution in cation-adjusted Mueller-Hinton broth. (**b**) Etest® Vancomycin results of the 56 *vanB* type outbreak VRE tested on MH agar using McFarland standard 0.5 and (**c**) and on Brain Heart Infusion agar using McFarland standard 2.0. The vancomycin MIC from microdilution in cation-adjusted Mueller-Hinton broth was included in Figures 1b and 1c for reasons of comparison. The dotted line in Figure 1a depicts the EUCAST breakpoint for clinical resistance of >4 mg/L and the dashed line depicts the CLSI resistance breakpoint of ≥32 mg/L (CLSI intermediate range of 8–16 mg/L, susceptible breakpoint for EUCAST and CLSI is ≤4 mg/L). The numbers on top of the bars display the number of isolates. Please notify that Etest® Vancomycin allows assessing a broader spectrum of antibiotic dilution concentrations than doubling dilutions assessed in MH broth microdilution.

For all isolates initial PCR results were confirmed; all were negative for *vanA* and 67 were positive for *vanB* leading to four which were negative for both genes. The vancomycin MIC distribution of the 67 VanB strains only is shown in Figure 1a revealing 34 strains (51%) with an MIC in the susceptible range of ≤4 mg/L according to CLSI and EUCAST criteria (see also below). All isolates showed a signal for IS*16* and almost all were positive for *esp* (n = 76; 97%) and *hyl*_*Efm*_ (n = 70; 90%).

### Sma*I macrorestriction in PFGE*

**Figure 2:**
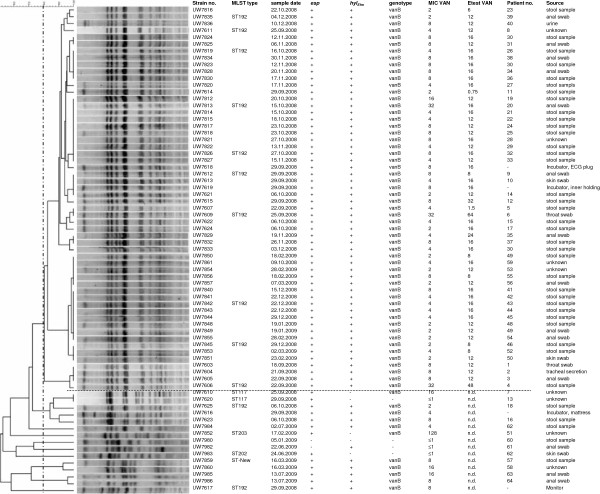
**Clonal relatedness of all investigated 71 enterococcal isolates as based on*SmaI*macrorestriction analysis in PFGE and subsequent phylogenetic analysis (Dice co-efficient using UPGMA clustering; BioNumerics v 6.5; settings: optimization 0.5%; tolerance 1.0%).** An 80% similarity line (dashed line) divides the group of the 56 related ST192 *vanB*-type outbreak isolates (above the horizontal dotted line) from the other 15 “non-outbreak” strains (below the dotted line). Legend: MIC VAN represents the MICs determined in microdilution in cation-adjusted Mueller-Hinton broth; Etest VAN is the value after 24 h readout on MH agar and using McFarland 0.5; n.d., not determined; ST-NEW represents a previously unrecognised MLST type with the pattern [9-1-1-1-12-7-1] which is a single locus variant of ST80.

To resolve a possible outbreak scenario we subjected all *E. faecium* isolates to *Sma*I macrorestriction analysis in PFGE. Altogether 56 isolates constituted a single cluster which was already obvious by visual inspection after clustering (optimization 0.5, tolerance setting 1.0) and when applying an 80% similarity score (Figure 2); these isolates are further designated as “outbreak strain type” or “outbreak isolates”. Isolates constituting the main cluster type derived from 52 neonatal patients; two isolates were from environmental sources and three originated from the same patient. The 15 non-outbreak strains revealed several smaller clusters or individual patterns, among them a pair of isolates with one isolate being *vanA/B*-negative (UW7620) and another one being *vanB*-positive (UW7610), the former being obviously the susceptible progenitor of the latter isolate having acquired a *vanB* gene cluster. We were unable to identify a *vanB*-negative progenitor isolate of the VanB type outbreak strain type.

### MLST analysis

Altogether ten strains representing the outbreak strain type were MLST typed. They were from different branches of the PFGE tree and all revealed the same sequence type ST192 (Figure 2). The two *van*-negative and *vanB*-positive isolates with the same PFGE pattern were ST117. Isolates representing individual PFGE patterns revealed ST203 (2 isolates) and ST192 (“non-outbreak” ST192 type). Results of molecular screening tests were congruent with the typing results. All isolates belonging to the outbreak strain (PFGE) type were *esp-* and *hyl*_*Efm*_-positive. Prevalence of both markers varied among the other 15 non-outbreak strains confirming the diverse strain background of these isolates.

### Comparison of the 56 outbreak isolates

Altogether 31 (55%) isolates of the outbreak *vanB* strain type had an MIC in the susceptible range of ≤4 mg/L (Figure 1) similar to results of the primary analysis using vancomycin disk diffusion. All 56 isolates were PCR-positive for genes *esp*, *hyl*_*Efm*_ and IS*16* (and *vanB*). In general, vancomycin Etest® MICs on MH agar were 1–2 doubling dilutions higher than in MH broth (Figure 1b,c). Etest® Vancomycin MICs on MH and BHI agar revealed two VRE (24/48 h) with a susceptible result of ≤4 mg/L only (Figure 1b,c). The 48 h Etest® Vancomycin MIC of VanB strains was commonly 0.5 - 1 doubling dilutions higher than after the 24 h readout, whereas it remained constant for susceptible reference isolates (Figure 1b,c). Performance of three chromogenic VRE agars to identify the 56 *vanB* type outbreak VRE was evaluated. On Brilliance^TM^ VRE agar, three isolates did not grow and two showed growths of small colonies only. The chromID^TM^ VRE agar showed a similar performance with three isolates that did not grow and one isolate that showed growth of small colonies only. On CHROMagar^TM^ VRE two isolates did not grow and another two grew with small colonies.

### *Determination of the* vanB *subtype*

The long PCR products amplified with DNA from nine ST192 outbreak isolates and three non-outbreak isolates (ST117; “non-outbreak ST192”; ST203) were subsequently digested with *BspH1/Dra1*. All but one showed a unique restriction pattern which is type-specific for *vanB2* subtype clusters ( Additional file 1: Figure S1). A single non-outbreak strain (ST203) possessed an identical pattern but an additional band.

### *Localisation of* vanB

**Figure 3:**
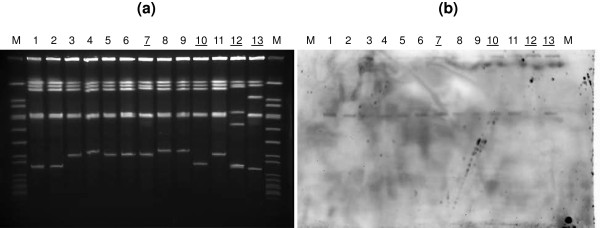
**Chromosomal localization of*vanB2*in*E. faecium*outbreak and non-outbreak strains.** (**a**) Genomic DNA digested with I-*CeuI* and resolved in PFGE; (**b**) Southern hybridisation with a labelled *vanB* probe. Underlined lane numbers designate “non-outbreak strains”. Legend: O, ST192 outbreak strain; NO, non-outbreak strains. M, *S. aureus* x *Sma*I; 1, UW7606(O); 2, UW7609(O); 3, UW7612(O); 4, UW7813(O); 5, UW7819(O); 6, UW7842(O); 7, UW7610 (NO, ST117); 8, UW7611 (O, ST192); 9, UW7835 (O); 10, UW7837 (NO, ST192); 11, UW7845(O); 11, UW7852 (NO, ST203); 12, UW7852 (NO, ST203); 13, UW7859 (NO, ST-NEW).

We performed different analyses with a set of nine MLST typed outbreak and four non-outbreak strains. To resolve the plasmid vs. a chromosomal localization we isolated the plasmids from 13 strains (see above; plus one additional ST203 isolate). The plasmid profiles of the nine outbreak strains were similar, the patterns of the four non-related strains differed; however, Southern hybridisations with a labelled *vanB* probe did not reveal any signal ( Additional file 2: Figure S2). Nevertheless, we performed PCRs for the three most common *E. faecium* plasmid types determined by their replicase genes, which were positive for *rep*(pRUM)(*rep*_*17*_ family according to [15]), *rep*(pRE25(*rep*_*2*_ family)), and *rep*(pLG1)(*repA_N* family, new class according to [16]) in all outbreak ST192 isolates and different for the other four isolates representing various sequence types (not shown in details). A single *Ceu-I* band of ca. 260 kb hybridised to the *vanB* gene probe in all PFGE lanes confirming the chromosomal localization of this determinant (Figure 3).

### Conjugation experiments

Altogether nine outbreak ST192 isolates (UW7606, UW7609, UW7611, UW7612, UW7813, UW7819, UW7835, UW7842, UW7845) were used as donors for in vitro filter-mating experiments and *E. faecium* 64/3 as a recipient. Only one mating experiment revealed transconjugants (UW7606) with a very low mating rate of 1,67 x 10e-8 (per recipient) and 7,14 x 10e-8 (per donor), respectively. A single transconjugant TC1 was investigated in details. TC1 became resistant to vancomycin but remained susceptible to erythromycin, gentamicin, ampicillin and ciprofloxacin. It was PCR-positive for IS*16* (recipient 64/3 is IS*16*-negative) most probably due to acquisition of a mobile *vanB* gene cluster which is commonly flanked by copies of IS*16*[5]. PFGE analysis of the donor, the recipient and the transconjugant followed by subsequent Southern hybridisation with a labelled *vanB* gene probe confirmed the clonal relatedness of the recipient and the transconjugant, the latter having acquired a mobile *vanB* gene cluster from the donor which integrated into the chromosome ( Additional file 3: Figure S3).

## Discussion

It has not been investigated in greater details until now to which extent VanB type strains may show a low expression of vancomycin resistance which could result in a distinct frequency of underestimation or underreporting of *vanB* type resistance in *Enterococcus* spp. in general. Results of our present study (Figure 1) and annual reports from our Focal laboratory for enterococci already demonstrated that low resistance expression among *vanB* type strains was prevalent in 10–25%, dependent on the method and breakpoints used [10,20]. It has to be noted again that in the present setting samples were screened non-selectively and selected enterococcal colonies were later tested for glycopeptide resistance phenotypically and genotypically. Most strategies to identify *vanA*- or *vanB*-type VRE start with selective enrichment or direct selection on screening plates which would certainly miss a substantial amount of strains showing low vancomycin resistance expression (see below). This general strategy may already pre-select the detectable wildtype *vanB* population. Nevertheless, non-selective screening was successful in this setting with premature infants lacking an established intestinal flora. In adult patients VRE account only for a certain percentage of all intestinal enterococci and non-selective screening would most likely be unable to select (by chance) for resistant variants [21]. Also real-time based screening assays targeting only the resistance genes *vanA* and *vanB* are not considered as a reliable alternative for screening since prevalence of *vanB* genes in non-enterococcal, intestinal colonizers complicates assay accuracy and leads to false positive test results [22-25]. The numbers are comparably similar in different parts of the world resulting in a generally low positive predictive value for VanB and the demand for a confirmation by culture based methods (with the problems as described above)[22,26,27]. In the light of our present study results, it cannot be excluded that the generally high false-positive *vanB* rate of real-time based genotypic assays could also result from the low performance of comparator assays to identify VanB strains with low vancomycin resistance levels and may thus to a lesser extent be attributed to *vanB* prevalent in non-enterocococcal species.

We assessed the performance of three commercially available chromogenic VRE selective agar plates in identifying the 56 VanB type outbreak *E. faecium* isolates with partly low level vancomycin resistance expression and compared it to results of Etest® Vancomycin MICs based on two different protocols. In general, MICs were 1–1.5 doubling dilutions higher on solid media (Etest®) than in liquid broth (Figure 1). In line with these results the three chromogenic agar media also performed comparably well in identifying *vanB* type VRE. A supposed better vancomycin resistance expression of *vanB* on solid media is an important finding, but observed here on a collection of admittedly similar isolates and thus cannot be extrapolated to the *vanB* VRE population in general.

At least two of the 56 outbreak VRE isolates originated from the patients’ surrounding and were clonally identical to the other patients’ isolates (Figure 2). Together with all the described infection control measures one might conclude that despite all rigid disinfection, infection prevention and training procedures, environmental VRE contamination remains a possible source for this ongoing VRE prevalence over three years. It is known for a long time that VRE carriers contaminate their direct surrounding and that VRE/enterococci are able to survive for weeks and months on these surfaces and patients commodities [28,29] and are thus able to spread to and colonise other patients. A common strategy to decolonise VRE patients and newborns is not established; however, results of recent experimental studies pinpoint towards strategies by eliminating VRE colonisation with probiotic competitor strains [30].

A number of European countries reported increasing numbers of colonisations and infections with VanB type VRE. Sweden having had extremely low overall VRE prevalence over the years experienced several clusters of colonisations and infections in hospitals in Stockholm recently [3,31]. Molecular analysis revealed distribution of *vanB2*-Tn*5382* subtype clusters located on pRUM-like transferable plasmids introduced into hospital-associated *E. faecium* strain types [7,8]. A similar resistance gene cluster was shown to be prevalent among various *E. faecalis* and *E. faecium* strains in 16 hospitals in Chile [32] although in general *vanA*-type resistance seemed most prevalent among Latin American VRE isolates (Peru, Colombia, Ecuador, Venzuela)[33]. A number of reports described *vanB* type VRE outbreaks in recent years among hospital patients in Spain and France representing European low VRE prevalence countries according to EARS-Net data [4,34-36]. Whereas the outbreaks in Spain remained at a local level (hospital outbreaks) and did not feed substantially the overall surveillance numbers; increasing VRE frequencies in France in 2008 are mainly attributed to a marked increase in a number of *vanB* outbreaks in the North of the country [4]. Similar rates are notified for Germany after 2008, at least for some federal states mainly in the South-Western part of the country [10].

During the ongoing outbreak also other non-outbreak *vanB* isolates were identified. It remains to be speculated if the identified *vanB2*-Tn*5382* cluster as part of an ICE is capable of spreading horizontally from strain to strain and integrate into a recipients’ genome. Molecular analysis revealed a similar cluster type and a chromosomal localization in all VanB strains. Resistance spread at a very low mating rate as assessed in vitro. Nevertheless, it was shown recently that vancomycin resistance genes are transferred successfully in vivo in mammal intestines and that rates could be several orders of magnitude higher than those determined in vitro [37-39].

Various expression levels of vancomycin resistance in *vanB* strains were known for some time. The level of *vanB* gene expression and the presence of a distinct *vanB* allele type (*vanB*1 to *vanB*3) could not be genetically linked. Grabsch et al. were unable in identifying mutational changes in unrelated *vanB*2 type VRE showing a various level of *vanB* gene expression [40]. Teicoplanin heteroresistance in *vanA* VRE strains was described recently and several genomic rearrangements and deletions within the *vanA* gene cluster elements and mutational changes within the two-component regulator genes *vanS* and *vanR* were identified in glycopeptide-heteroresistant strains, but these changes were not (experimentally proven) functionally linked to the described heteroresistance phenotype [41-43]. We did not investigate the molecular background of different *vanB* resistance expression in the isolates investigated here. Nevertheless, MICs were reproducible in repeated experiments.

## Conclusions

We analysed an ongoing high prevalence of *vanB* type *E. faecium* in a neonatal ICU over a period of several years; ca. 80% (n = 56) of isolates collected during a key period of 10 months belonged to a single outbreak strain type. Enterococcal isolates were assessed non-selectively and about half of the isolates appeared phenotypically susceptible to vancomycin with MICs ≤4 mg/L. This suggests that a certain amount of VanB strains may show a low expression of vancomycin resistance which results in an unknown frequency of underestimation or underreporting of *vanB* type resistance in *Enterococcus* spp in general. It is tempting to speculate that this phenomenon may directly support ongoing and increasing prevalence of (unrecognized) *vanB* VRE prevalence among the clinical setting. The comparably high false-positive *vanB* rate of real-time based genotypic assays may thus only be partly attributed to *vanB* prevalent in non-enterocococcal species but could also simply result from the low accuracy of comparator assays to identify VanB strains with low expression of vancomycin resistance.

## Competing interests

The authors declare that they have no competing interests.

## Authors’ contributions

HMJ and RZ co-ordinated, supervised and evaluated the primary diagnostics of the clinical samples and consulted the staff at the neonatal ICU regularly. UG determined the spectrum of antimicrobial susceptibilities by broth microdilution and performed all confirmatory PCR screenings. CF performed all Etests®, chromogenic agar assays, PFGE and Southern hybridisation experiments. IK, WW and GW supervised the confirmatory diagnostic and molecular typing experiments and interpreted and analyzed the results. GW, IK and RZ wrote the manuscript.

## Supplementary Material

Additional file 1 Figure S1. *vanB2* subtype determination. Long PCR products with DNA from outbreak and non-outbreak strains were subsequently digested with *BspH1/DraI*. Underlined lane numbers designate “non-outbreak strains”. Legend: O, ST192 outbreak strain; NO, non-outbreak strains. M, Gene Ruler 100bp Plus (Thermo Fisher Scientific); 1, UW7606(O); 2, UW7609(O); 3, UW7612(O); 4, UW7813(O); 5, UW7819(O); 6, UW7842(O); 7, UW7610 (NO, ST117); 8, UW7611 (O, ST192); 9, UW7835 (O); 10, UW7842 (O); 11, UW7845(O); 12, UW7852 (NO, ST203) [UW7859 (NO, ST203) did not reveal a long PCR product; not shown]. Click here for file

Additional file 2 Figure S2. Plasmid patterns of *vanB2 E. faecium* outbreak and non-outbreak strains. (a) Undigested plasmid patterns resolved in 0.8% agarose gel; (b) Southern hybridisation with a labelled *vanB* probe. Underlined lane numbers designate “non-outbreak strains”. Legend: O, ST192 outbreak strain; NO, non-outbreak strains; M, Roche Size Marker III, DIG-labelled (for orientation purposes only); 1, UW7606(O); 2, UW7609(O); 3, UW7612(O); 4, UW7813(O); 5, UW7819(O); 6, UW7842(O); 7, UW7610 (NO, ST117); 8, UW7611(O, ST192); 9, UW7835 (O); 10, UW7845(O); 11, UW7852 (NO, ST203); 12, UW7859 (NO, ST203). Click here for file

Additional file 3 Figure S3. (a) *Sma*I-digested genomic DNA resolved in PFGE and (b) Southern hybridisation with a labelled *vanB* probe of a *vanB* type *E. faecium* donor strain UW7706, a vancomycin-susceptible recipient *E. faecium* 64/3 and a *vanB*-positive transconjugant 1. Legend: M, *S.aureus* x *Sma*I. D, donor strain UW7706; R, recipent strain 64/3; T, transconjugant UW7706x64/3 TC1. Please note that the *vanB* positive band in lanes D and T refers to a double band. Click here for file
