# Supplementary material for: Vancomycin-resistant vanB-type Enterococcus faecium isolates expressing varying levels of vancomycin resistance and being highly prevalent among neonatal patients in a single ICU
Source: Antimicrob Resist Infect Control. 2012 May 30;1:21. doi: 10.1186/2047-2994-1-21 (PMC3533821; doi:10.1186/2047-2994-1-21)
Supplement: Additional file 1 Figure S1. — vanB2 subtype determination. Long PCR products with DNA from outbreak and non-outbreak strains were subsequently digested with BspH1/DraI. Underlined lane numbers designate “non-outbreak strains”. Legend: O, ST192 outbreak strain; NO, non-outbreak strains. M, Gene Ruler 100bp Plus (Thermo Fisher Scientific); 1, UW7606(O); 2, UW7609(O); 3, UW7612(O); 4, UW7813(O); 5, UW7819(O); 6, UW7842(O); 7, UW7610 (NO, ST117); 8, UW7611 (O, ST192); 9, UW7835 (O); 10, UW7842 (O); 11, UW7845(O); 12, UW7852 (NO, ST203) [UW7859 (NO, ST203) did not reveal a long PCR product; not shown]. [file 2047-2994-1-21-S1.ppt]

## Slide 1
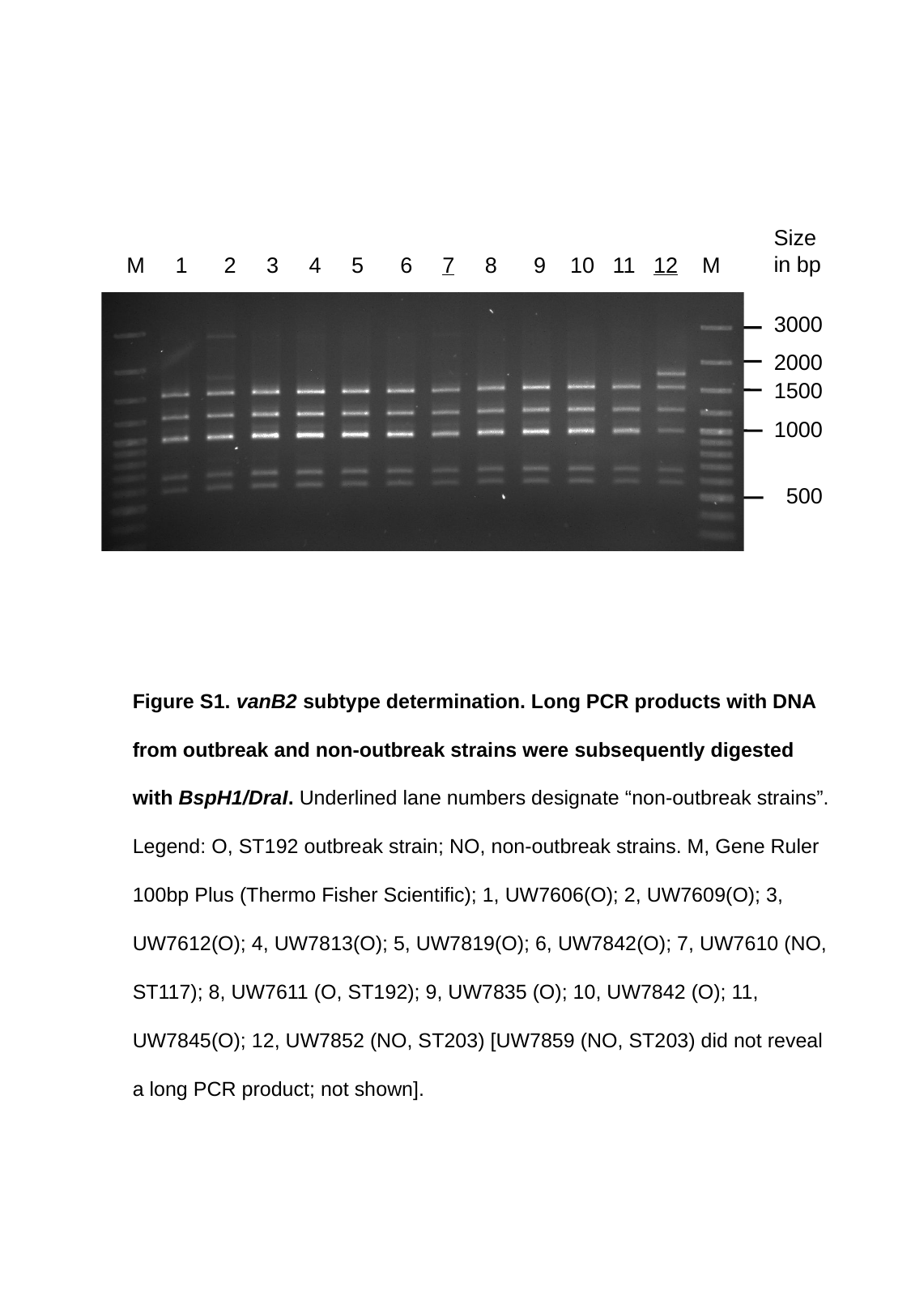

Size
in bp
M 1 2 3 4 5 6 7 8 9 10 11 12 M
3000
2000
1500
1000
500
Figure S1. vanB2 subtype determination. Long PCR products with DNA from outbreak and non-outbreak strains were subsequently digested with BspH1/DraI. Underlined lane numbers designate “non-outbreak strains”. Legend: O, ST192 outbreak strain; NO, non-outbreak strains. M, Gene Ruler 100bp Plus (Thermo Fisher Scientific); 1, UW7606(O); 2, UW7609(O); 3, UW7612(O); 4, UW7813(O); 5, UW7819(O); 6, UW7842(O); 7, UW7610 (NO, ST117); 8, UW7611 (O, ST192); 9, UW7835 (O); 10, UW7842 (O); 11, UW7845(O); 12, UW7852 (NO, ST203) [UW7859 (NO, ST203) did not reveal a long PCR product; not shown].
