# Supplementary material for: Vancomycin-resistant vanB-type Enterococcus faecium isolates expressing varying levels of vancomycin resistance and being highly prevalent among neonatal patients in a single ICU
Source: Antimicrob Resist Infect Control. 2012 May 30;1:21. doi: 10.1186/2047-2994-1-21 (PMC3533821; doi:10.1186/2047-2994-1-21)
Supplement: Additional file 2 Figure S2. — Plasmid patterns of vanB2 E. faecium outbreak and non-outbreak strains. (a) Undigested plasmid patterns resolved in 0.8% agarose gel; (b) Southern hybridisation with a labelled vanB probe. Underlined lane numbers designate “non-outbreak strains”. Legend: O, ST192 outbreak strain; NO, non-outbreak strains; M, Roche Size Marker III, DIG-labelled (for orientation purposes only); 1, UW7606(O); 2, UW7609(O); 3, UW7612(O); 4, UW7813(O); 5, UW7819(O); 6, UW7842(O); 7, UW7610 (NO, ST117); 8, UW7611(O, ST192); 9, UW7835 (O); 10, UW7845(O); 11, UW7852 (NO, ST203); 12, UW7859 (NO, ST203). [file 2047-2994-1-21-S2.ppt]

## Slide 1
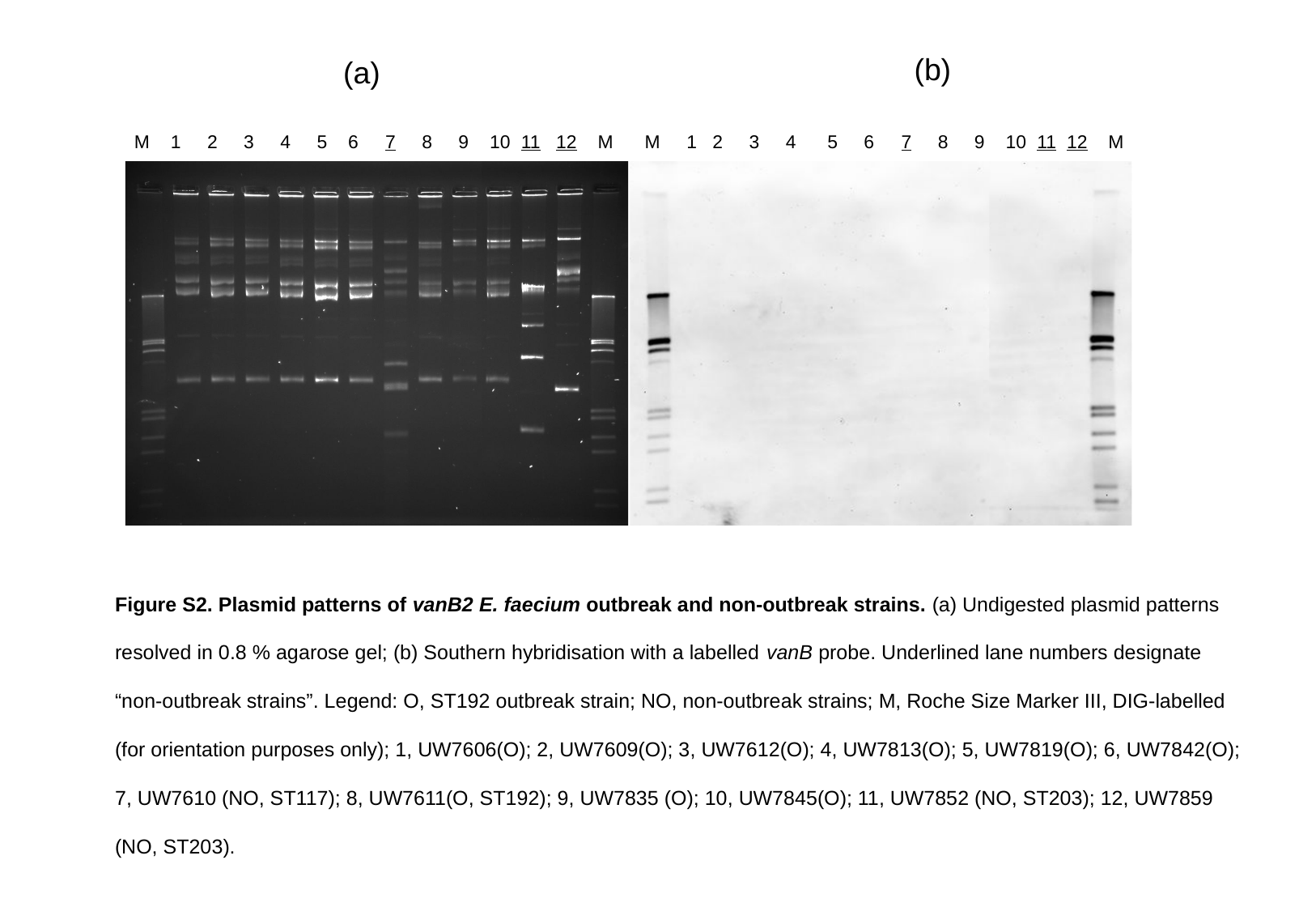

(b)
(a)
M 1 2 3 4 5 6 7 8 9 10 11 12 M M 1 2 3 4 5 6 7 8 9 10 11 12 M
Figure S2. Plasmid patterns of vanB2 E. faecium outbreak and non-outbreak strains. (a) Undigested plasmid patterns resolved in 0.8 % agarose gel; (b) Southern hybridisation with a labelled vanB probe. Underlined lane numbers designate “non-outbreak strains”. Legend: O, ST192 outbreak strain; NO, non-outbreak strains; M, Roche Size Marker III, DIG-labelled (for orientation purposes only); 1, UW7606(O); 2, UW7609(O); 3, UW7612(O); 4, UW7813(O); 5, UW7819(O); 6, UW7842(O); 7, UW7610 (NO, ST117); 8, UW7611(O, ST192); 9, UW7835 (O); 10, UW7845(O); 11, UW7852 (NO, ST203); 12, UW7859 (NO, ST203).
