# Supplementary material for: Vancomycin-resistant vanB-type Enterococcus faecium isolates expressing varying levels of vancomycin resistance and being highly prevalent among neonatal patients in a single ICU
Source: Antimicrob Resist Infect Control. 2012 May 30;1:21. doi: 10.1186/2047-2994-1-21 (PMC3533821; doi:10.1186/2047-2994-1-21)
Supplement: Additional file 3 Figure S3. — (a) SmaI-digested genomic DNA resolved in PFGE and (b) Southern hybridisation with a labelled vanB probe of a vanB type E. faecium donor strain UW7706, a vancomycin-susceptible recipient E. faecium 64/3 and a vanB-positive transconjugant 1. Legend: M, S.aureus x SmaI. D, donor strain UW7706; R, recipent strain 64/3; T, transconjugant UW7706x64/3 TC1. Please note that the vanB positive band in lanes D and T refers to a double band. [file 2047-2994-1-21-S3.ppt]

## Slide 1
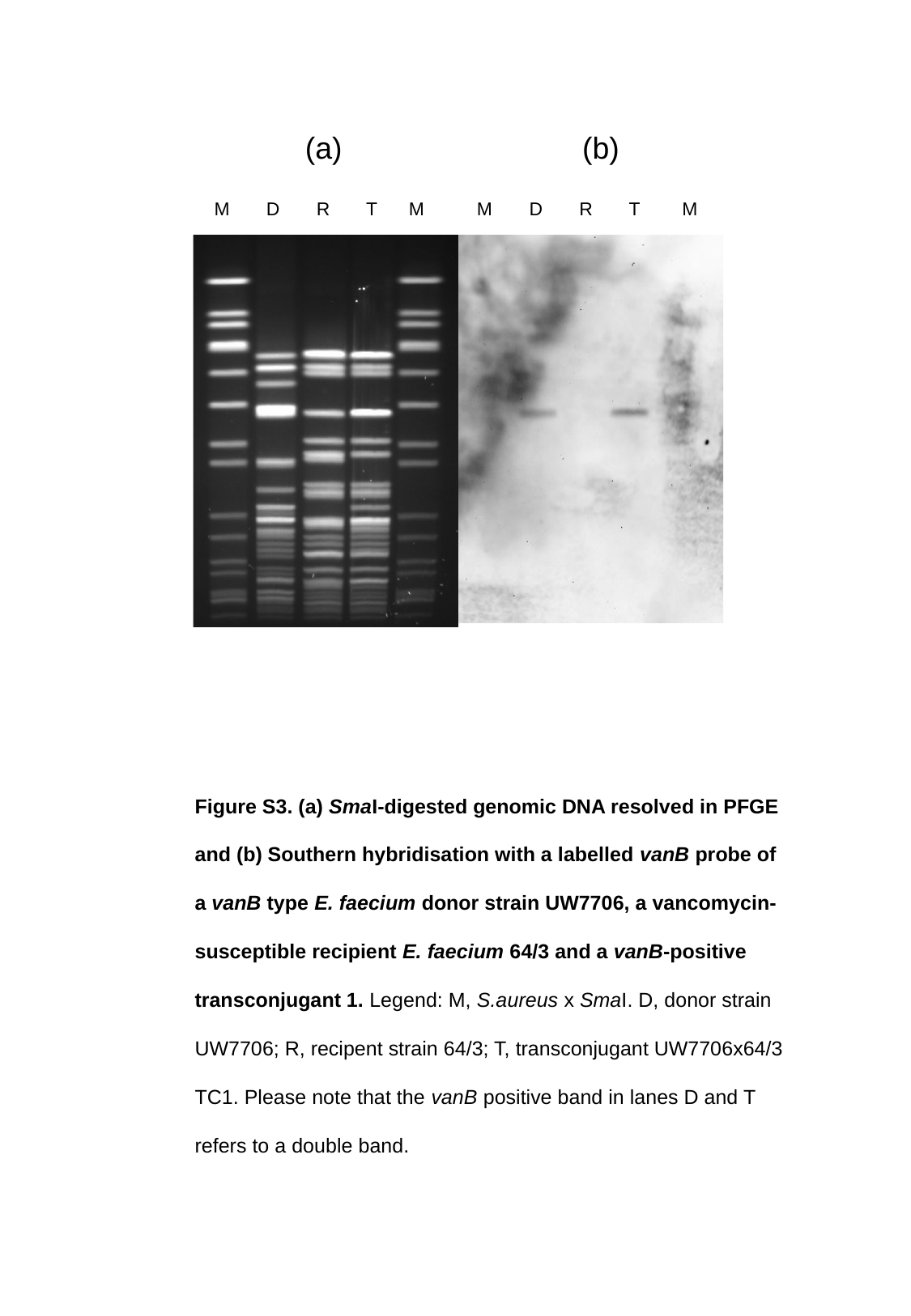

(a)
(b)
M D R T M M D R T M
Figure S3. (a) SmaI-digested genomic DNA resolved in PFGE and (b) Southern hybridisation with a labelled vanB probe of a vanB type E. faecium donor strain UW7706, a vancomycin-susceptible recipient E. faecium 64/3 and a vanB-positive transconjugant 1. Legend: M, S.aureus x SmaI. D, donor strain UW7706; R, recipent strain 64/3; T, transconjugant UW7706x64/3 TC1. Please note that the vanB positive band in lanes D and T refers to a double band.
